# Who Cares for Those Who Take Care? Risks and Resources of Work in Care Homes

**DOI:** 10.3389/fpsyg.2018.00314

**Published:** 2018-03-14

**Authors:** Caterina Gozzoli, Diletta Gazzaroli, Chiara D’Angelo

**Affiliations:** Department of Psychology, Università Cattolica del Sacro Cuore, Milan, Italy

**Keywords:** care professions, Care Homes, risk and resources, malaise, well-being

## Abstract

Over the years – due to the aging population, the process of corporatisation and a demand for a higher quality of services – professionals who work in Care Homes have been exposed to an increasing risk of physical and emotional malaise because of the number of challenges they’ve been asked to manage. Given these factors, there is a growing interest in the study and understanding of professions in geriatric care settings. In the literature there is a prevalence of quantitative studies offering an overview in terms of indicators – at the individual or group or organizational level – concerning the potential development of situations of professional malaise. Conversely, there is a lack of qualitative studies exploring the risk and protection factors. For this reason, in this study we decided to use a qualitative approach to explore “more up close” this kind of organizational context and to keep together the different levels in systemic terms in order to identify – according to professionals’ perceptions – resource factors (in order to leverage these aspects) and fatigue factors (to identify them and treat them). Three Italian Care Homes were involved and the interview’s sample was composed of 45 professionals – 15 nurses, 30 total Patient Care Assistants (PCAs) and Auxiliary Care Assistants (ACAs), of these, 17 males and 28 females, with an average age of 43 years (*SD* = 0.78) – selected using a sampling of maximum variability. From the analysis of the materials there seem to be four profiles of the professionals involved. Implications to ensure a functional human resource management are discussed for the purpose of promote the well-being of the various professionals, and, as a result, an increasing quality of service.

## Introduction

The present study aims to offer a contribution to the understanding of the care professions that work in Care Homes [in Italy, Residenze Sanitarie Assistenziali (RSA)]^1^ because due to the complex challenges they face they are likely to experience various forms of physical and emotional malaise.

First evidence related to the attention for care professions concerns the incremental aging of the Italian population: 200,000 non-self-sufficient seniors are guests in residential structures, 2.5 million live with their families, but 4.7 million seniors would opt to live in residences if their quality of life improved (Censis, 2015). Furthermore, the increased age of the guests welcomed in Care Homes determines a physical and mental health situation characterized by co-occurrence of illnesses, psychological and behavioral instability, fragility (Sanchez et al., 2015; Cooper et al., 2016), and therefore the work required of operators is focused on the preservation of the remaining capacity, the management of chronic illness and assistance in activities of daily living (Abrahamson et al., 2009; Spinelli et al., 2016). Another element to consider is the process of corporatisation that increasingly impacts Italian care settings, implying that professionals are supposed to be able to optimize time, resources, and costs. At the same time, there is a demand by family members and guests for a higher quality of services (Cigoli et al., 2003; Leininger, 2005) and a consequent increase in the degree of competitiveness, pressure within this sector and demands in terms of emotional and physical investment that professionals must deal with.

Given these factors, there is a growing interest in the study and understanding of professions in geriatric care settings. In the literature (see Kennedy, 2005; Jeon et al., 2012; Sanchez et al., 2015; Cooper et al., 2016) there is a prevalence of cohort, cross-sectional and comparative studies that offer an overview in terms of indicators – at the individual or group or organizational level – concerning the potential development of situations of professional malaise. Studies that examined the individual dimension have shown that a lack of sense of control and mastery over one’s working conditions and fear of making mistakes represent risk factors for operators (Duquette et al., 1995; Estryn-Behar et al., 2011). It is also true that the ability to exercise decision-making autonomy with respect to the assumption of one’s role can represent not only an important protection factor, but also a stimulus in terms of performance and skills development (Willemse et al., 2012). Studies on elements related to the group show that poor levels of coordination and integration between teams and roles, and difficulties with interpersonal relationships (Estryn-Behar et al., 2011) are factors that affect the well-being of the people who make up the working team. This is in line with the assumption that social support from colleagues acts as a “buffer” against the negative effects of the work on one’s health and well-being (Viswesvaran et al., 1999; Deelstra et al., 2003). Additionally, the professionals who work in Care Homes very often have to negotiate with the role expectations of guests and their families, as well as with those of colleagues and the organization (Abrahamson et al., 2009). In fact, the transition to a care home for a family is much more than a change in location, and often family members pour out all their anxieties and fears related to this stage of the family cycle on operators. More infrequent are the studies on elements related to the organizational level (Kane et al., 2007; André et al., 2014). They showed the influence that culture and climate have on operator comfort. More precisely, some studies have shown that elements like a low degree of individual responsibility attributed to the individual operator, a lack of open and clear communication, a rigid leadership, the lack of spaces and channels for discussion are more likely to be associated with episodes of stress, job dissatisfaction, and poor quality of work (Clarke et al., 2002). Similarly, a correlation has been demonstrated between organizational cultures and climates oriented toward encouraging and promoting the processes of empowerment, communication and participation and the influence these have on the quality of life in Care Homes both for operators and for guests (André et al., 2014). To this must be added the uncertainty and insecurity related to the continuous changes that affect the healthcare world, and the quantity and quality of information made available to professionals may contribute positively or negatively to well-being. In summary, analyzing the literature we can see that most works are quantitative research focused mostly on just one analysis level. Conversely, there are fewer qualitative studies that extensively explore the risk and protection factors. For these reasons, this study therefore aims to “give a voice” to the operators exploring efforts and resources that they perceive and represent to be a priority for their profession in order to further the knowledge of this phenomenon and identify lines of action in support of the professionals and organizational effectiveness. In light of our experience researching and working with organizations, it was decided to approach the complexity of these contexts by adopting a psycho-sociological approach to the study of professional roles (Lewin, 1951; Kelly, 1955; Barus-Michel et al., 2002; Gorli et al., 2012; Gozzoli et al., 2015; Gozzoli, 2016a,b). This means simultaneously keeping in mind and considering three different levels:

(1) The individual level (the stories and the personal and professional experiences of the participants).
(2) The group level (the operation of the work group, relationships with colleagues).
(3) The organizational level (the operation of the organization as a whole and its organizational culture).

Within this approach, maintaining the connection between these levels is crucial because, while on the one hand the personal and professional identity of the individual questions the social and organizational context, on the other hand the organizational and social context redefines the questions posed by the individual in terms of limitations and possibilities.

## Materials and Methods

### Aims and Scope

Through a qualitative study developed in three Italian Care Homes,^2^ the purpose of this paper is to identify and explore resource factors (in order to leverage these aspects) and fatigue factors (to identify them and treat them) in the perception of professionals. In fact, with this work we want to invite reflection that is useful in promoting the protection of the well-being of professionals working in these contexts, and consequently, the quality of the service offered.

### Sample and Measures

The data collection process through semi-structured interviews involved different professionals operating in three Lombard Care Homes. For the study, it was decided to involve nurses, Patient Care Assistants (PCAs) [in Italian, Operatore Socio-Sanitario (OSS)] and Auxiliary Care Assistants (ACAs) [in Italian, Ausiliario Socio-Assistenziale (ASA)] because given the nature of the service provided these were the professional categories found to be most stressed by the issues in question. More precisely, 45 professionals from the three Care Homes were interviewed, selected using a sampling of maximum variability that made it possible to have a representative sample in terms of proportion of personnel working in the three facilities: 15 nurses, 30 total PCAs and ACAs. Of these, 17 males and 28 females, with an average age of 43 years (*SD* = 0.78).

### Data Analysis

In line with org goals, we conducted a paper-and-pencil content analysis following the phenomenological interpretative approach (Smith et al., 1999; Smith and Osborn, 2003; Smith, 2004; Brocki and Wearden, 2006), which aims to understand the experiences and explore the process of construction of meaning that individuals use to understand the subjective perspective, and taking into account the socio-cultural context in the data interpretation process.

We worked with a hierarchical categorization system combining a top-down and a bottom-up logic. To categorize the text, with a top-down logic we defined three macro-categories (that is, “individual history,” “group and relationships,” and “organization”) consistent with the psycho-sociological approach on study of professional roles. After this, in order to identify micro-categories for each one of the three macro-categories, we analyzed the text portions considered with a bottom-up logic. We combined these logics for two reasons:

(1) In the one hand, it is known that different research projects exploring professional context (Stokols, 2000; Scaratti et al., 2009; Pietarinen et al., 2013; Gozzoli et al., 2014; Guglielmetti et al., 2014; Tamanza et al., 2016) agree about the importance of considering individual level, group level and organizational level (top-down logic);
(2) On the other, is not yet so well established which specific indicators should be considered on each of the three levels of analysis (bottom-up logic).

Consequently, we combined two kind of content analysis. As defined by Hsieh and Shannon (2005), we applied the “directed content analysis” (codes are defined before and eventually, during data analysis) and the “conventional content analysis” (categories and names for categories flow from the data). Precisely, during the top-down phase, we applied the directed content analysis looking for the three main macro-categories defined according to our theoretical background. During the bottom-up phase, we applied the conventional content analysis in order to discover the specific micro-categories characterizing professionals and organizations involved in the study.

After these two phases, we started an interpretative process in order to explain the relationship between macro- and micro-categories.

Data analysis was conducted by three independent reviewers (the agreement was calculated for each of the under-pairs of judges CG and DG, CG and CD, and DG and CD and after that the mean value we calculated). Inter-rater reliability was good (Cohen’s *K* = 83%) and was calculated using ComKappa software (Cohen, 1960; Robinson and Bakeman, 1998). Cases of disagreement were considered and discussed until consensus was reached.

Next will be presented the findings that were common to the majority of the operators of the three Care Homes involved in the study.

## Results

We will present the key overall findings that arose from the interviews with respect to the objectives of this study. Following will be presented the recurring and cross-cutting elements with regard to the three different levels considered – individual, group, and organizational – looking at resource versus risk. Then we will analyze their different articulation.

### “My History” – Individual Level

From the interviews, it emerges how the professional histories of the participants are distributed in a balanced way between two main and different *motivations*: intrinsic or extrinsic.

In the case of a choice linked to an intrinsic motivation for entering the profession, participants talk about situations that “triggered something” – a family illness, dissatisfaction with previous professional roles perceived as “empty and alienating” the desire to “be useful” – and have thus led to a career in a care home.

“I graduated in accounting, then I did my civil service, in contact with the elderly, from there I became interested in this world.”

ACA Operator

“I made this change when my grandmother passed away. I still had this thing inside me of wanting to stay with the elderly... I went to that school because I wanted to stay with seniors.”

ACA Operator

“Ever since I did an internship in a care home, even when I was taking a nursing course over 30 years ago, I really loved working with senior citizens. Inside me there was always the desire to end up in a care home...so...it wasn’t by chance.”

Nurse

With regard to extrinsic motivations, however, these situations are not directly related to the emergence of a desire or a propensity to care, but rather circumstances that influenced the decision to work in a care home, like loss of a job, economic necessity, or difficulty in entering the desired profession.

“This friend of mine knew that there was a work grant offered by the Lombardy Region and that you could take advantage of this grant to take an ACA course, even those people who had lost their jobs in the textile industry. Many women like me lost their jobs... So that’s it, that’s how it all started. There was nothing inside me pushing me to become an ACA....”

ACA Operator

“I was not born for this profession.... I chose this job because I needed to work. If I didn’t have to be here I would have left.”

PCA Operator

“I was a seamstress for a company that produced clothing... We were at home receiving unemployment, the region offered this ACA course for free... I needed to work. There was no other work so I went into this field.”

ACA Operator

Correlated with these considerations, two different *representation of the professional role* has emerged. From the analysis of the collected materials it emerges that representing one’s role with a social value, as a choice based on an inner desire and an individual propensity to care for others are factors of protection. Moreover, the relationship of care and sense of self-efficacy emerges as a significant source of satisfaction. In contrast, in cases where the role is represented as a fall-back or experienced as a temporary professional transition, what emerge is the perception of oppression and a lack of financial and social recognition. These elements are the most common risk factors for these professionals.

The representation of the role is also relevant with respect to how the workload is experienced. In fact, in cases where the role is experienced as a personal choice, the fatigue that often emerges in managing work plans is balanced by focusing attention on pursuing the primary objective of “ensuring the well-being of the guest.” However, it should be pointed out that also in these cases we can find a risk component if there is a sense of not being able to provide care in the time and manner deemed most suitable by the users. In cases where the role is seen as a fallback, however, the workload is perceived as being physically and psychologically tiring, the experience that emerges is that of being “exploited and unrecognized,” of being machines that have to “produce numbers and results.”

“You’re always running against the clock, but you have to do it for the sake of the elderly... So sure, sometimes you’re sorry that you can’t stop to chat when they need it even if you know that you’re behind schedule....”

ACA Operator

“I’m alone so I have to move to cover three wards...but they want the therapies done by 9.00 am because then there are the activities...but in the meantime, they continue to call you from one room to another.”

Nurse

In terms of feelings of fatigue a view shared across the board by the various professionals – no matter if the role is represented as a choice or as a fall-back – is the perception of distance between education, the refresher courses offered to them and daily work practices. It emerges that all the professionals involved in this research state they feel the need to receive training courses aimed at making them able to respond adequately to the challenges and changing affecting care world. Another factor that emerges that is experienced as a critical issue, and partly connected to the previous one, is the lack of being able to consider any professional development.

### “The Others and the Relationships” – Group Level

As for relationship dynamics, three nuclei have been examined around which revolves the majority of exchanges: the elderly, colleagues, and management.

*The elderly and their central role in the caregiving*, which for most professionals is a source of gratification. However, one must not forget that the relationship with the elderly can also pose a potential risk factor with regard to the management of physical and emotional fatigue. In fact, the interviews show that if one becomes “too” close to the pain of Others there is a risk of being overwhelmed. Conversely, if one remains “too” distant the risk is rejecting it and not processing it in a manner that is conscious and functional for “caring for the Other.”

“The relationship with guests is nice, I feel good, they always give me a lot.”

ACA Operator

*Colleagues* are an important relational source fuelled by a strong sense of belonging within the same professional category. However, outside the same professional category, they are often perceived as distant due to hierarchical structures and to the years of experience in the field. The words of the respondents in most cases seem to characterize the relationships between colleagues with different roles as “difficult” or “poor.”

“There is a bit of division...some small groups. If one gets too concerned with these things it can undermine the work a little. It can undermine pretty much everything.”

PCA Operator

“In the ward, if we all help each other the group works better... I’ve even had some colleagues who told me that, for certain things, they wouldn’t do very much... They told me: ‘From this point on... if I don’t feel like it, I won’t do it’.”

ACA Operator

“The main difficulties regard the discrepancies between the nursing team and the ACA group; interference of things unsaid or not taken into account purely with regard to healthcare management, injury identification.”

Nurse

In terms of climate perceptions, two main issues seem to emerge from the analysis, shared transversely, which can affect the quality and the perception of support among professionals: “invasions/confusion of role” and communication problems.

The invasions of role – referring to the overlap that can be created when one professional category invades that of other professionals in terms of duties or powers – can lead to the creation of intergroup conflicts. In this regard, there are three main types that emerge on the part of the professionals. The first is of those who, fulfilling their role in proactive terms, feel “compelled” to facilitate work processes even for other colleagues and so some activity is performed in terms of cooperation to carry out functions that do not necessarily fall within their role. A second type is linked with an experience of lack of recognition of skill and professional ability with respect to the work of colleagues or other professionals in one’s own field. Finally, a third type is that of having an even greater workload when it is necessary to carry out functions that do not necessarily fall within one’s work responsibilities.

If we don’t have baths, [we] can do weights. If I have the night shift I get the weights ready for him, so he finds everything ready.

ACA Operator

“They have us ACAs administering therapies and we shouldn’t, but we do them anyway. We are forced to do them, like small medications....”

ACA Operator

This subject is inevitably linked to another important organizational issue: responsibility. Clearly assuming one’s role in terms of proactivity and flexibility with respect to protocols and functions, on the one hand, requires the individual to make a conscious choice to respond to what he or she does as a professional with specific skills. On the other, it implies a willingness on the part of management (representing the organizational mandate) to give its professionals a certain degree of autonomy and professional trust.

Similarly, communication, which is perceived as problematic, represents a critical element in the protection of work processes and intra- and inter-group relationships. Emblematic with respect to this dimension is the time of handoff – the summary of health conditions, needs, and problems of each guest that is passed on at the end of each shift to those beginning the next one – in which all the people who work in the same ward or floor meet to exchange information. From the data, it emerges that this activity is perceived as being done hastily and with one-way communication between the person handing off (the nurse) and the listener, or only with communication exchanges between colleagues of the same role without effective integration of the information.

“During a handoff, it happened that we were talking about a guest and we talked about them in front of the nurses. The nurse on duty told me: “Listen, can’t you stay quiet, because we’re doing the handoff.” But this is part of the handoff. Because if I’m talking about a particular problem of the guest....”

ACA Operator

“...these things are not feasible, the handoff is missing things...handoffs are missing completely, there are no procedures, protocols. These things are missing, that, in my opinion, are critical to communicate with each other.”

PCA Operator

The final nucleus emerging is that of relational dynamics experienced with *management*. Here again emerges a dual representation, shared by all. When management is perceived to be close and able to listen it is represented a resource that can be relied on. Otherwise, when it is perceived and represented as being absent and distant from the everyday activities of operators it constitutes a risk factor. Often there is an overlap between the organization and management that is seen as something far away, “other” with respect to everyday work.

With regard to differences between nurses and assistants, the analysis of the data revealed that for the most part there are no significant discrepancies except when it comes to management. In fact, interviews with nurses found as a potential source of risk dealing with the Health Director (regarding medical aspects) and the Organization Director (regarding assistance and organizational aspects) if these two roles are not aligned in their management of the professionals and in how guests should be assisted. This factor does not emerge from the ACA and PCA interviews since, providing assistance, they refer solely to the Organization Director.

### “Care Homes: Cure and Care” – Organizational Level

As for the issue of organizational culture, for all occupations and facilities there is a perception of a presence of two cultures: the *culture of care* versus the *culture of efficiency*. The first emerges from the declaration of values that states the focus on the guest, on promoting a welcoming atmosphere and on the attempt to promote a “family” dimension (“this is the guest’s ‘home”’). The second culture emerges from the frenzy that characterizes the management of the work and the need to balance the issue of quality of care and the relationship with that of the “quantity” of operational requests worthy of an “assembly line.” These two cultures, while in theory they should be simultaneous and complementary, are perceived by most of the professionals in terms of a choice, and what separates the professionals of these organizations is the way in which they react to this choice. Some of them, in fact, become polarized by choosing either to focus on care or on efficiency. Others, on the other hand, remain in the middle with a consequent feeling of confusion.

With regard to the work performed, a resource that has been identified is the multiplicity of roles and duties, the division of guests into wards by type of needs and health status, the presence of established practices of direct management of patients at different times and places (“everyone knows what to do and when to do it”).

“When establishing the Personal Health Plan (PHP) everybody is present: nurses, health director, operators, physical therapist, activities director, all involved in the discussion... That’s a pretty good opportunity because there we set goals for the next 6 months, all together, and absolutely if we didn’t do that our communication would be horrible. That’s a good thing even though there’s so much more that we should say.”

ACA Operator

At the same time, it is evident that these elements are perceived as resources that are not fully exploited because they are often carried out in a “hasty” manner, resulting in the tendency for there to be moments of meetings or informal exchanges between groups of “single professional categories,” used as opportunities to let off steam and discuss things. Also underlined is the difficulty in respecting the daily schedule.

“Every once in a while, we [nurses] meet to talk and a bit of everything comes up. Sometimes they are constructive, sometimes not. Sometimes they are positive, sometimes not. We talk about guests and issues regarding guests, but a bit of everything comes up.”

Nurse

“Meetings among operators are few, few, and few. That is, when you are on a shift together there is a chance to say something. But rarely is there a possibility for everybody to get together.”

ACA Operator

We can say that the two organizational cultures should not be interpreted as positive/negative. They are both equally necessary to ensure the quality of service and the well-being of guests and professionals. What may cause a difference in terms of impact is the way in which the two mandates can be read in terms of proximity or distance by each professional, in light of their professional and personal history.

## Discussion

From the analysis of the materials and the interrelation between the different elements there seem to be four profiles of the professionals involved, and therefore these profiles are crucial also to ensure a functional human resource management to ensure the well-being of the various professionals, and as a result, an increasing quality of service (Shippee et al., 2015). More precisely, it is possible to distinguish four different profiles based on the intersection of:

• Personal motivation that led to the choice of a role in a context like that of the care home;
• Role representation;
• Relationships with colleagues, the elderly, and the management;
• Organizational culture.

### Well-Balanced Profile

In the case of a well-balanced profile, profession is a “choice,” i.e., backed by a strong intrinsic motivation and the desire to care for Others, with a strong and significant investment in the relationship of care. At the same time, professionals show a good understanding of the fatigue (physical and psychological) related to proximity with the elderly. There is a good ability to have a close relationship with the elderly and with colleagues, one feels a “part” of an organization and for this reason is oriented toward the establishment of a communicative working group that is open to dialog, the fatigue and workload seem to be more manageable thanks to sharing and the ability to ask for support to colleagues and to the management. This type of profile not only is able to balance the different organizational requests – ensuring quality of service – but is also less exposed to the risk of malaise.

### Idealizing Profile

Professionals with an idealizing profile show an intrinsic motivation to invest in one’s role and relationship of care that can lead to not seeing the importance of time and space to recognize, identify, and discuss one’s physical and (especially) mental and emotional fatigue. With respect to colleagues, what emerges is a tendency to worry about how colleagues are doing their work and “taking upon oneself” parts of others’ work for fear that they are not being done “as they should” (quote from an interview). There is a certain degree of fatigue/impatience with respect to some of the reasoning behind the organizational culture and management’s requests, perceived as an “obstacle” to the relationship with the elderly. In this case the risk of malaise is high due to an excessive emotional closeness with the elderly and the hesitancy to request support from colleagues, and if not properly managed can lead to burnout (and therefore the impairment of the service itself).

### Well-Adapted Profile

In the case of well-adapted profile the professional choice was “unintentional,” i.e., the job was taken for reasons of chance/fall-back/need/by force. What is found is an increased emotional distance with respect to the closeness in the care relationship, and in the medium/long-term this can be protective with regard to the emotional overload that these professions are exposed to. Although sometimes there is no proactivity in the interpretation of the role there’s a good capability of interaction with colleagues and management. There is a good ability to manage work times and spaces (and thus guarantee service quality).

### Retreated Profile

With regard to retreated profile what is found is an emotional detachment with respect to the care relationship, lack of gratification in performing one’s role and therefore a widespread difficulty (or, as it has been defined in some cases, an impossibility) of imagining a career path and professional growth. Furthermore, where there is no sense of belonging (if not within circumscribed micro-groups of people with the same professional role). The prescriptive execution of the role leads to difficulties with communication and the management of conflicts. Organizational culture is perceived as excessive in its requests to professionals. In the medium/long term this can lead to demotivation and alienation from the elderly and colleagues (and therefore negatively affecting the quality of service).

In light of these four profiles the importance can be seen of defining and sharing the “perimeter” within which professionals must perform their roles and the degree of responsibility that is asked of them. At the individual level – besides requiring a considerable awareness of one’s professional skills – it involves finding the “right balance” within which to assume one’s role in terms of proactivity without delegitimising colleagues and the different responsibilities in the organization. At a micro/group level it requires the development of a work environment that allows a greater level of integration among roles, skills, professional cultures, and hierarchical lines. Finally, in terms of macro/organization it requires an investment in professional trust with respect to all roles in the organization and employing a paradigm not based on control. Another important nucleus in all four profiles is related to the presence/absence of meaningful relationships: what emerges across the roles and organizations is the weight that relationships or lack thereof can have with respect to the well-being of professionals. Similarly, the presence (and use) of work teams plays a significant role for the care home operators. The possibility to have times and places to meet not only for purely bureaucratic aspects but also to have the opportunity to “suspend” the action and the frenzy that characterizes everyday life is presented as an indispensable requirement to be able to start a process of true integration among parties and visions in play in a multidisciplinary context like the care home. Another fundamental relationship, both when perceived and experienced as being close by and when represented as distant (or absent), is the one with management. A risk factor in terms of emotional and professional “resistance,” in fact, is represented by the perception of a Management that is not willing to listen and recognize not only the hardships related to time management, bureaucracy, emotional experiences, and physical fatigue, but also to the identification of professional differences and perspectives on the guest.

The combination of these factors would seem to be crucial in promoting experiences and representations of being or not in a protective organizational context that is able to reconcile the split between two aspects that are simultaneously present in the care home: care – which is based on a relationship – and performance – which is based on the standardization of protocols.

### Limitations and Future Directions

Reasoning on the tool used in this research (although the interview has been very rich with all the participants), it only grasps verbal and individual elements. In future study on this topic, it would be interesting to combine the use of tools that can capture elements at different levels. In this sense, an interesting integration could be provided by ethnographic observations. In fact, observations will make it possible to discover where and when in daily practices are placed the dimensions of fatigue and resources. Moreover, considering the complexity and sensitivity of the topic, graphic symbolic tools will allow to know psychic dimensions less aware that can play an important role in terms of well-being and malaise (see for example, Karnieli-Miller et al., 2017). In future study, it would be also interesting to involve management roles and integrate the different perceptions on the issue.

According with our aims we kept the focus on the key overall findings; however, future studies (with a larger sample) could also investigate whether there are differences between different professional groups. Finally, a larger sample of professionals and organizations, could also allow to discover if risks and resources factors emerged in this study will need integrations.

## Conclusion

As pointed out at the beginning, the professionals that operate in places like Care Homes are strongly exposed to physical and psychological risks, therefore the possibility of realizing activities focused on preventing dissatisfaction, working on strengthening protective factors and reducing risk factors is critical to ensuring optimal levels of quality of life that protect professionals.

From this first study, it is already possible to identify some interesting operational issues and possible fall-outs. Among these, addressing the operators’ perception of distance from management; explore and have managers reflect on the complexity of the care home to understand how to communicate and support their employees in addressing the issues of care and effectiveness.

With reference to the topic of loneliness and fatigue in maintaining one’s role, identify in terms of organizational sustainability which solutions to enact to give the opportunity to manage the exchanges among professionals or how to use existing ones in a different way. Such action could be useful, not only to prepare spaces/moments of coordination among different professionals and hierarchies to establish more effective communication protocols, but also to support and accompany an exchange and emotional reworking, and identification of practices that promote autonomy in one’s role.

Another note to keep in mind in terms of analysis and potential for possible operational impact is the obstacle of professional growth. Define which subjects and skills professionals and the organization deem to be a priority for training programs, refresher courses and supervision, and just as important, for assessment of effectiveness.

In summary, what could come from such an analysis?

• Promoting greater awareness of the workers in order not to risk burnout and the emergence of heated conflicts.
• Understanding what aspects to focus on to reduce the risk of demotivation and routinisation of professional practices.
• Identifying possible individual and/or group areas to support these operators.
• Identifying and supporting the resources that allow the service to be more effective and efficient.
• Understanding how the organization as a whole can be put in a position to better address both internal and external changes that it encounters with increasing frequency.

At the end of our work, we can say that organizations are asked to create and support a “care culture” not only for the users and their families but also – and, in provocative terms, we would say primarily – for the professionals who are daily asked to take care of the others.

## Ethics Statement

This study was carried out in accordance with the recommendations of ‘Comitato Etico della Ricerca Psicologica, Dipartimento di Psicologia, Università Cattolica del Sacro Cuore’ with written informed consent from all subjects. All subjects gave written informed consent in accordance with the Italian Legislative Decree June 30, 2003, No. 196. The written informed consent is reported below: ‘I agree to the proposal to participate in the research study. My agreement is an expression of a free decision, not influenced by promises of economic benefits or otherwise, nor from obligations to the principal investigator of the study. I am aware of being free to withdraw from the study at any time I want. Moreover, I am aware that I’m not supposed to give any reasons for my decision to withdraw from the study. I was given the opportunity to ask questions about the aims and methods of the study and my rights as a participant in the research. I understood all the information and explanations that I have been given and I had enough time to consider my participation in this study. According to the Italian Legislative Decree June 30, 2003, No. 196, I agree that my personal data will be used for specific research purposes within the limits and in the manner explained in the information document.’ The protocol was approved by ‘Comitato Etico della Ricerca Psicologica.’

## Author Contributions

CG: scientific supervision of the research project. DG and CD: researcher of the project.

## Conflict of Interest Statement

The authors declare that the research was conducted in the absence of any commercial or financial relationships that could be construed as a potential conflict of interest.

## Footnotes

1 According to Italian Presidential Decree January 14, 1997, Care Homes in Italy are “places that offer to those who are not self-sufficient, elderly and otherwise, having physical, mental, sensory or mixed pathologies that cannot be treated at home, a level of medical, nursing, and rehabilitation care accompanied by a high level of protection and lodging assistance, modulated according to the care model adopted by the regions and autonomous provinces.”

2 We focused on three Care Homes in the Lombardy Region (North of Italy).
